# Maternal mortality ratio in Jiangsu Province, China: recent trends and associated factors

**DOI:** 10.1186/s12884-021-03897-0

**Published:** 2021-06-25

**Authors:** Donghua Li, Chengxiao Yu, Ci Song, Weiqing Ning, Yan Xu, Huan Ge, Song Lin, Wenjie Zhou, Yajun Lu, Xudong Wang, Zhibin Hu, Yuan Lin, Jie Wu

**Affiliations:** 1 State Key Laboratory of Reproductive Medicine, Department of Obstetrics and Gynecology, the First Affiliated Hospital of Nanjing Medical University, Jiangsu Province Hospital, Jiangsu Women and Children Health Hospital, Nanjing, 210036 China; 2 Department of Epidemiology, Center for Global Health, School of Public Health, Nanjing Medical University, Nanjing, 211166 China; 3 Jiangsu Key Lab of Cancer Biomarkers, Prevention and Treatment, Collaborative Innovation Center for Cancer Personalized Medicine, Nanjing Medical University, Nanjing, 211166 China; 4 Department of Women and Children, Jiangsu Provincial Commission of Health, Nanjing, 210008 China; 5 Department of Maternal, Child and Adolescent Health, School of Public Health, Nanjing Medical University, Nanjing, 211166 China

**Keywords:** Maternal mortality ratio, Trends, Regional differences

## Abstract

**Background:**

In recent years, births to older mothers and multiparous mothers have increased rapidly with the change of birth policy in China. And mothers of advanced age are more likely to have maternal complications and poor birth outcomes. We aimed to estimate the recent trends and underlying risk factors of maternal mortality.

**Methods:**

In this systematic assessment, we used data from the National Maternal and Child Health Routine Reporting System (2013–2018), Jiangsu Provincial Maternal Mortality Surveillance System (2017–2018), the Integrated National Mortality Surveillance System (2018), City Statistical Yearbooks (2018), City Health Statistical Yearbooks (2018). The factors associated with maternal mortality ratio (MMR) were explored using the stepwise regression analysis and cluster analysis.

**Results:**

The MMR maintained at low levels between 2013 and 2016 and there was a slight increase in maternal mortality after 2016 in Jiangsu province. With the implementation of the China’s universal two child policies, the percentage of multiparous mothers ascended from 34.2% (95% confidence interval (CI) = 34.1–34.3%) in 2013 to 51.4% (95% CI = 51.3–51.6%) in 2018 (beta = 3.88, *P* < 0.001). Consistently, the percentage of advanced maternal age (≥ 35) increased from 8.4% (95% CI = 8.4–8.5%) in 2013 to 10.4% (95% CI = 10.3–10.4%) in 2018 (beta = 0.50, *P* = 0.012). And we found that the percentage of multiparous mothers and advanced maternal age among maternal deaths were higher than all pregnant women (*P* < 0.001). In the stepwise regression analysis, four risk factors were significantly associated with maternal mortality ratio (primary industry of gross domestic product (GDP), rate of delivery in maternal and child health hospital, rate of cesarean section and rate of low birth weight). As the results derived from cluster analysis, the relatively developed regions had lower preventable maternal mortality ratio (43.5% (95% CI = 31.2–56.7%) *vs.* 62.6% (95% CI = 52.3–72.0%), *P* = 0.027).

**Conclusions:**

Since the universal two child policy has been associated with changes in health related birth characteristics: women giving birth have been more likely to be multiparous, and more likely to be aged 35 and over. This somewhat magnifies the impact of differences in economic development and obstetric services on MMR. The findings based on prefecture level data suggest that interventions must target economic development, the health system and maternal risk factors in synergy. These approaches will be of great benefit to control or diminish environmental factors associated with preventable deaths and will effectively reduce MMR and narrow the gap among the different regions.

**Supplementary Information:**

The online version contains supplementary material available at 10.1186/s12884-021-03897-0.

## Introduction

Maternal mortality ratio (MMR) has been a priority area for the global health and development community at least since the Nairobi Safe Motherhood Conference in 1987 [1–3]. Since the United Nations proposed a 75% reduction in the global MMR between 1990 and 2015 (Millennium Development Goal 5, MDG5) [4], governments worldwide have defined their own goals and strategies for MMR reduction accordingly. As one of the few Countdown countries to have achieved the goal, China has pushed down the maternal mortality ratio at an annualized rate of 6.5% per year, one of the fastest decreases in the world. The national maternal mortality ratio fell from 111.0 per 100,000 livebirths in 1990 to 21.8 per 100,000 livebirths in 2015 [5]. Since then, to ameliorate the nation’s stagnant population growth, shrinking workforce, and ageing population, the universal two-child policy were enacted in October 2015[6]. Women of reproductive age with a previous delivery were targeted by the policy, and who would have been allowed to have a second child. The policy would have stimulated births to older mothers, a higher risk population for many pregnancy complications and poor birth outcomes [7, 8]. The accumulation of older mothers may possibly give rise to a rebound in MMR after achieving MDG5. However, to the best of our knowledge, there were little research published on the trends in maternal mortality in China since 2015 in the medical literature.

Jiangsu province is located along the east coast of China (116°18'-121°57' E and 30°45'-35°20' N), and plays an important role in the downstream of Yangtze River Basin, covering an area of 10.72 km^2^ and had total population of 80.5 million in the year 2018. The implementation of the Yangtze River development strategy has contributed to the overall development of Jiangsu, which enabled it to be one of the most developed provinces in China. In 2018, the gross domestic product (GDP) of Jiangsu province reached 9259.5 billion yuan, accounting for about 10.3% of the China’s GDP. In recent years, the level of development presents a ladder-like distribution from north to south affected by factors such as resources, economy along the Yangtze River and government-oriented [9]. Such differences in development levels may lead to the degree and trends for maternal mortality remain heterogeneous at the county level [3]. Therefore, we aimed to estimate the underlying risk of maternal mortality that is affected by relevant key determinants based on the county level.

In this study, we described recent trends of MMR and explored whether the increase of MMR was associated with the accumulation of mothers of advanced age. Then, the characteristic analysis of maternal mortality and related factors based on the county level is helpful to further explore the risk factors related to maternal mortality at the macro level. We aimed to to inform strategies for further improvements in maternal health and reduce MMR in the different regions of Jiangsu Province.

## Methods

### Overview of study design and analysis

We started by describing the trend of MMR from 2013 to 2018 in Jiangsu province, the fifth largest population province in China, and analyzing the increase in the percentage of multiparous mothers and older mothers from 2013 to 2018 due to the implementation of the China’s universal two child policies. We chose 2017–2018 as the period for analyzing geographic variations of MMR because the policies impact had stabilized by then. We described the differences in maternal mortality in each region of Jiangsu province over the same period, and explored the factors associated with MMR using a stepwise regression analysis. Finally, we informed strategies for further improvements in maternal health in the different characteristic regions of Jiangsu province.

### Data sources

First, we obtained data on the number of livebirths, maternal deaths, the percentage of multiparous mothers and advanced maternal age (≥ 35) in Jiangsu province between 2013 and 2018 from the National Maternal and Child Health Routine Reporting System [10]. Second, we used data from the individual level maternal death information records in Jiangsu province between 1 January 2017 to 31 December 2018 from the Provincial Maternal Mortality Surveillance Systems (161 maternal deaths) [11]. Maternal deaths are defined as deaths of women who are “pregnant or within 42 days of termination of pregnancy, irrespective of the duration and site of the pregnancy, from any cause related to or aggravated by the pregnancy or its management but not from accidental or incidental causes” [12]. Third, we obtained the causes of death data for women aged 15–49 years for 2018 according to the Integrated National Mortality Surveillance System [13]. Fourth, data on resident population, rate of population growth, gross domestic product (GDP) per capita in counties and districts of Jiangsu province in 2018 were extracted from City Statistical Yearbooks [14]. Fifth, data on the number of livebirths and maternal deaths (2017–2018), the number of health care card, the number of prenatal examination, the number of postpartum visit, the number of delivery in public general hospital or maternal and child health hospital, the number of maternal system management, the number of cesarean section, the number of moderate to severe anemia, the number of high risk for prenatal screening, the number of maternal at risk, the number of low birth weight, the number of high birth weight, the number of preterm birth and the number of stillbirth in counties and districts of Jiangsu province in 2018 were extracted from City Health Statistical Yearbooks [15].

### Data quality management

In order to ensure the accuracy of monitoring data, the investigators must visit departments related to maternal mortality surveillance, such as the Vital Statistical System, Household Registration Department of the Ministry of Public Security, healthcare institutions, and departments of family planning and crematories to verify the number of live births, maternal mortality and causes of death and make prompt corrections to underreporting or misdiagnoses of death cause [16].

To collect information for maternal death review, an investigation was conducted by at least one specialty trained obstetrician from the county/district maternal and child health hospital. For deaths inside the hospital, hospital records and other related information were collected by document reviewing or personal interviewing. For deaths outside the hospital, information was collected by household visit. This sort of investigation focused on personal/family background, overall situation during pregnancy, onset of any disease and corresponding treatment, course of delivery, the date of termination of pregnancy, and the date of maternal death. Subjects of investigation were the husband, family members and relatives, midwife, obstetrician, nursing staff and others close to the deceased woman. Upon completion of the investigation, the investigation form, medical examiner reports, medical records, and autopsy reports for each case were submitted to the review committee at the county/district level of the surveillance area [16].

### Maternal death review

If the death may have been averted by one or more changes in the healthcare system related to clinical care, facility infrastructure, public health infrastructure and/or patient factors, a death was considered preventable. For each case classified as potentially preventable, a determination was made regarding the ways in which the death might have been averted [17].

According to the World Health Organization (WHO) recommendation [18], each case of maternal death was reviewed by the review committees at the county/district, provincial, and state levels. After receiving the mortality report, the committee at the county/district level conducted the preliminary review: (1) to check the completeness and accuracy of the report and request additional information for the report if needed and (2) to determine causes of death, identify preventable areas and associated factors, and suggest interventions. The review committees at the provincial and state level re-examined the results submitted by the subordinate committee, undertook the joint review for contentious cases, and made the final decision based on the majority of opinions. Causes of death were encoded in accordance with ICD-10.

### Statistical analysis

We used histogram to describe the order of causes of women death aged 15–49 and 20–34 (2018), the percentage of multiparous mothers (2013–2018), and the percentage of advanced maternal age (2013–2018). The administrative map of Jiangsu province were was drawn “maptools” R package. The factors associated with MMR were explored using a stepwise regression analysis. We divided each region of Jiangsu province into two groups through cluster analysis. Welch's t-test was applied for comparison of differences between two groups. Statistical analyses were performed using R software (version 3.5.0). The significance level was set at *P* < 0.05 and *P* values were given for two-sided tests.

## Results

### The necessity to focus on MMR

**Fig. 1:**
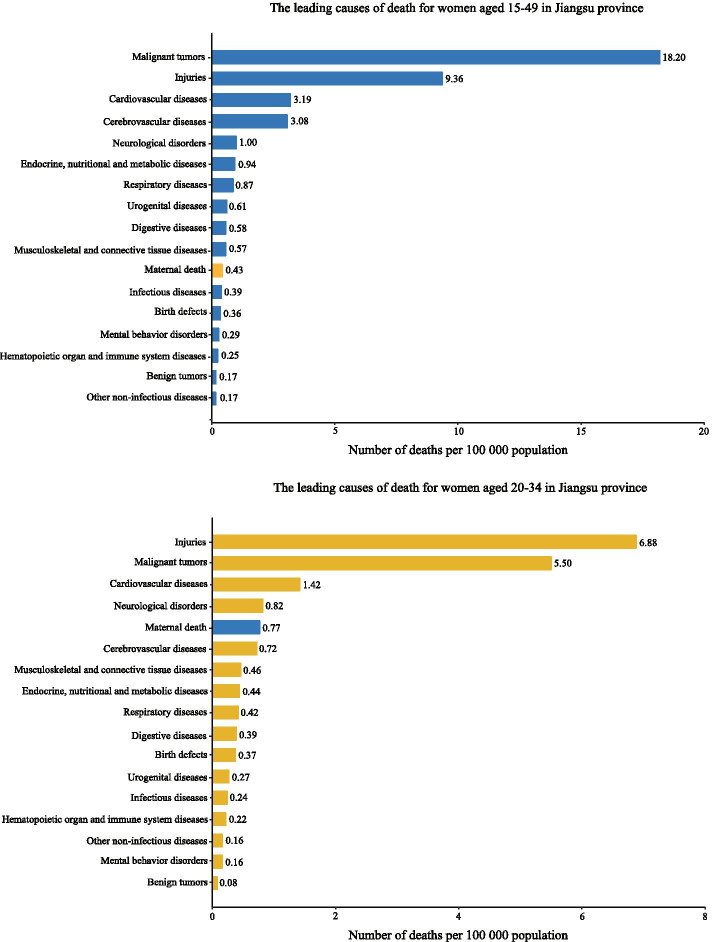
The leading causes of death for women of reproductive age in Jiangsu province, 2018

According to the 2018 Jiangsu Provincial Center for Disease Control and Prevention's cause-of-death database, maternal mortality is the eleventh leading cause for women death aged 15–49 and the fifth leading cause for women death aged 20–34 in Jiangsu provincial (Fig. 1). According to the data of Jiangsu Provincial Statistical Yearbooks and the National Maternal and Child Health Routine Reporting System, the changes in maternal mortality ratio were stable and maintained at low levels between 2013 and 2016 (4.99, 4.65, 4.64, 4.47/100000 livebirths, respectively), signaling that Jiangsu provincial has achieved MDG5. However, there was a slight increase in MMR after 2016, the MMR was 10.42/100000 livebirths in 2017 and 9.83/100000 livebirths in 2018 (Figure S1).

### The impact after the policy began to take effect

**Fig. 2:**
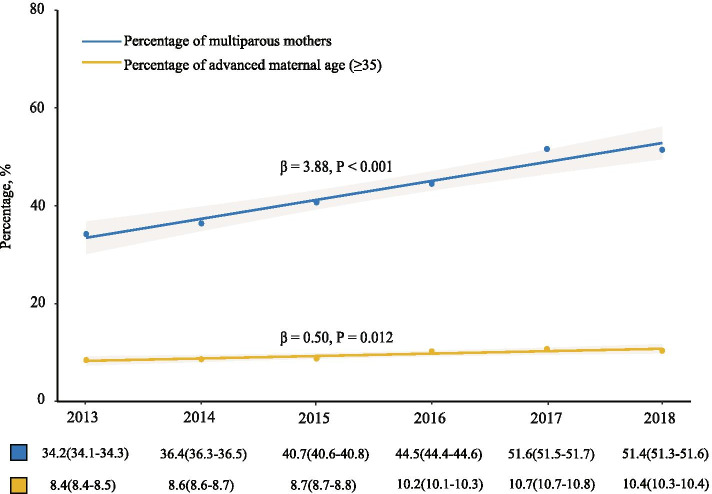
Secular trends of the percentage of multiparous mothers and advanced maternal age (≥ 35) during 2013 and 2018 in Jiangsu Province. The label under the figure: Percentage, % (95% confidence interval (CI))

With the implementation of the China’s universal two child policies, the percentage of multiparous mothers ascended from 34.2% (95% confidence interval (CI) = 34.1–34.3%) in 2013 to 51.6% (95% CI = 51.5–51.7%) in 2017, and reach a plateau in 2018 (51.4%, 95% CI = 51.3–51.6%). Consistently, the percentage of advanced maternal age increased from 8.4% (95% CI = 8.4–8.5%) in 2013 to 10.7% (95% CI = 10.7–10.8%) in 2017, and reach a plateau in 2018 (10.4%, 95% CI = 10.3–10.4%). There is a significant upward trend in the percentage of multiparous mothers (beta = 3.88, *P* < 0.001) and the percentage of advanced maternal age (beta = 0.50, *P* = 0.012) (Fig. 2). In more detailed data, we find that the percentage of multiparous mothers variation between all pregnant women (51.5%, 95% CI = 51.4–51.6%) and maternal deaths (79.5%, 95% CI = 72.3–85.3%) was substantial (*P* < 0.001) in 2017–2018. The percentage of advanced maternal age among maternal deaths (26.1%, 95% CI = 19.6–33.7%) was significantly higher than the percentage of advanced maternal age among all pregnant women (10.5%, 95% CI = 10.5–10.6%) (Figure S2).

### To formulate improvement strategies according to local conditions during the period of stabilization after the policy takes effect

In this part, we described the geographical differences in MMR in Jiangsu province under the general trend that policy changes have led to an increase in the percentage of multiparous mothers and the proportion of older mothers, and analyzed the characteristics of each region in Jiangsu province.

**Fig. 3:**
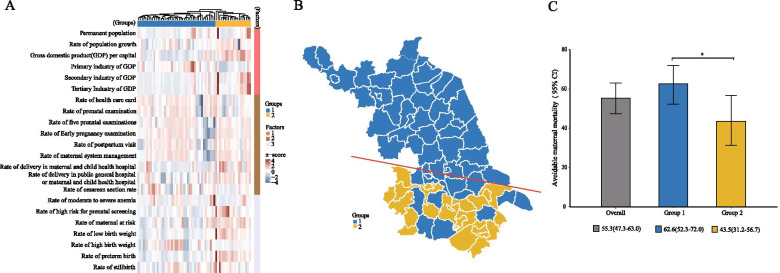
The clusters of counties and districts in terms of the related risk factors (Jiangsu province, 2018). A The counties and districts Jiangsu province was divided into Group 1 (blue) and Group 2 (yellow) according to the cluster analysis; All the risk factors involved in the three aspects (Factor 1: the level of economic development; Factor 2: the health systems and health consciousness; Factor 3: maternal risk factors) for cluster analysis by z-score; B The geographical distribution of groups in Jiangsu province; C The preventable maternal mortality in Group 1 and Group 2 regions. *: *P* < 0.05. The label under the figure: preventable maternal mortality, % (95% confidence interval (CI))

Combined with the administrative map of Jiangsu province, we found that the MMR (2017–2018) in the southern region was slightly lower than that in the northern region. In the stepwise regression analysis, four risk factors were significantly associated with maternal mortality ratio, including the level of economic development (primary industry of gross domestic product (GDP), *P* = 0.003), the health systems and health consciousness (rate of delivery in maternal and child health hospital, *P* = 0.094; rate of cesarean section, *P* = 0.015) and maternal risk factors themselves aspects (rate of low birth weight, *P* = 0.003) (Table 1). We included all the risk factors involved in the above three aspects for cluster analysis (Fig. 3A). As the results derived from cluster analysis, Jiangsu province was divided into Group 1 and Group 2 according to the clustering of different characteristic factors (Fig. 3B). In addition, the relatively developed regions had lower preventable maternal mortality ratio, 43.5% (95% CI = 31.2–56.7%) in the Group 2 regions *vs.* 62.6% (95% CI = 52.3–72.0%) in the Group 1 regions (*P* = 0.027) (Fig. 3C). Comprehensive analysis of the relevant objective risk factors for maternal death, we found that Group 2 regions have a higher level of economic development, better health resources and awareness. However, pregnant and lying-in women in the Group 2 regions had a higher proportion of risk factors than the women in the Group 1 regions (Table S1).

**Table 1:** Stepwise regression analysis of objective factors related to maternal death

Factors	Coef	95% CI^a^		P*
Primary industry of GDP	1.24E-06	4.29E-07	2.06E-06	0.003
Rate of delivery in maternal and child health hospital	-1.28E-04	-2.78E-04	2.26E-05	0.094
Rate of cesarean section	3.32E-04	6.64E-05	5.97E-04	0.015
Rate of low birth weight	3.52E-03	1.29E-03	5.74E-03	0.003

^*^ Significance level: *P* < 0.1

^a^*CI* Confidence interval

## Discussion

Based on data retrieved from the National Maternal and Child Health Routine Reporting System between 2013 and 2018, we have described the timing and trends in maternal mortality in Jiangsu province. Our findings concur with previous analyses suggesting that China is making substantial progress towards achieving the target of 75% reduction in MMR [19–21]. However, MMR had rebounded slightly after a rapid decline. We observed a noteworthy increase after the universal two policy began to take effect, the percentage of multiparous mothers and advanced maternal age had risen to varying degrees. Based on the above characteristics and the different characteristics of economic development level, health system and risk factors in different counties and districts of Jiangsu province, we further found the underlying risk difference of maternal mortality in different regions that is affected by relevant key determinants. And there are regional disparities in preventable maternal mortality in the different regions of Jiangsu Province.

MMR has always been a key indicator when evaluating a country/region's socioeconomic development. China's spending on health as a percentage of GDP has been rising, and a high level of investment in health infrastructure has kept pace with development [22]. Meanwhile, increased household income may have increased economic opportunities for hospital delivery [23], and reduced delays in care-seeking around the time of birth. From 2013, families could have two children if one parent was an only child [24]. The universal two-child policy of 2016 has been associated with changes in health-related birth characteristics: women giving birth have been more likely to be aged 35 and over, and more likely to be multiparous. There is growing evidence that advanced maternal age is associated with a range of adverse pregnancy outcomes, including maternal near miss, severe obstetric complications, even maternal death [8, 25–27]. Therefore, the increase in maternal age of the targeted multiparous mothers contributes to the growing MMR after the universal two child policy took effect.

China’s current disparities and imbalanced progress in health are alarming [28]. Although Jiangsu is a highly developed province in the economy, medical treatment and education when compared with other areas in China, the imbalance of medical development and economic between different regions still exists. It has been widely recognized that maternal mortality ratio in a specified region is influenced by socioeconomic status in addition to the level of healthcare systems. In China, 80% of health resources were concentrated in relatively developed cities and large urban hospitals [29, 30]. Relatively developed regions had more abundant medical and human resources, and the number of medical technicians was much higher than that of relatively remote areas [30]. Risk factors associated with maternal mortality are usually controlled through environmental improvements or interventions, which means that a reduction in maternal mortality is essentially a reduction in preventable maternal mortality. The preventable maternal mortality rate in a specified region reflects both the quality of the quality of life of women and its obstetric services in that region. Many reports indicate that the percentage of preventable maternal deaths varies around the world, including United States (40%) [17], Japan (37%) [31], South Australia (44.4%) [32], Nigeria (70.2%) [33], and Brazil (90%) [34]. In contrast, the preventable maternal mortality ratio in Jiangsu was higher than that in high-income countries, especially in less developed areas (Group 1) of the province.

Our findings reveal that the factors associated with maternal mortality also differed by region. In relatively developed regions (Group 2) that enjoyed developed economies, rich health resources, easy transport and high accessibility of health services, most women preferred maternal and child health hospital delivery. However, less developed areas had fewer high-risk women than relatively developed region, which was probably due to fewer hospital visits of the women in less developed areas where health resources were relatively scarce. Once pregnant women develop high-risk status, the referral system in Jiangsu province will refer them to relatively developed region. Thus, for the Jiangsu province government, it is important to improve the knowledge/skills of individuals/families and health professionals in medical institutions, irrespective of where they live [16]. At the same time, attention should be paid to the improvement of health awareness and medical conditions in Group 1 areas, and improve the capacity of Group 1 and 2 regions to deal with and treat dangerous pregnant women.

Our study has some limitations: firstly, the early abortion-related maternal deaths outside the hospital were likely to be underreported, especially in the regions with a weak maternal and child healthcare system, although the Provincial Maternal Mortality Surveillance Systems covers the entire period of pregnancy and has unified quality control measures. Secondly, the study was conducted in Jiangsu province, which is a highly developed province in the economy, education and medical treatment in China. Whether the findings are applicable to other areas, for example western China areas, needs to be assessed.

In summary, the advanced maternal age deserves more attention with the changes in China's two child policies. Regional differences exist in maternal mortality rates and related factors. Improvements in the healthcare or economy would go a long way in reducing preventable maternal mortality and minimizing MMR and the regional gaps within Jiangsu province. Overall, It is necessary for the health sector to formulate strategies to reduce MMR in accordance with the actual situation in the different regions of Jiangsu Province. The findings also suggest that if economy, culture and healthcare improvements were made in developing country/region, maternal mortality rates would be significantly reduced.

## Supplementary Information

**Additional file 1: Figure S1.** Trends of MMR in Jiangsu province from 2013 to 2018. **Figure S2.** The percentage of multiparous mothers and advanced maternal age (≥35) of all pregnant women and maternal deaths from 2017 to 2018 in Jiangsu province. ***: *P*<0.001. The label under the figure: Percentage, % (95% confidence interval (CI)). **Table S1.** Objective risk factors for maternal death were different between the two groups.

## Data Availability

We obtained the data from the Jiangsu Women and Children Health Information Center. The ownership of the data belongs to Jiangsu Women and Children Health Information Center. Researchers who meet the criteria for access to confidential data can contact Yan Xu (xuyanblue@sina.com) at the Jiangsu Women and Children Health Information Center to request the data. Statistical Yearbooks (City Statistical Yearbooks; City Health Statistical Yearbooks) datasets are publicly available. The datasets used for the current study are also available from the corresponding author on reasonable request.

## References

[1] Smith SL, Rodriguez MA (2016). Agenda setting for maternal survival: the power of global health networks and norms. Health Policy Plan.

[2] Gao Y, Zhou H, Singh NS, Powell-Jackson T, Nash S, Yang M (2017). Progress and challenges in maternal health in western China: a Countdown to 2015 national case study. Lancet Glob Health.

[3] Liang J, Li X, Kang C, Wang Y, Kulikoff XR, Coates MM (2019). Maternal mortality ratios in 2852 Chinese counties, 1996–2015, and achievement of Millennium Development Goal 5 in China: a subnational analysis of the Global Burden of Disease Study 2016. Lancet.

[4] United Nations. Implementation of the United Nations Millennium Declaration. Report of the Secretary General; 2002

[5] Collaborators GBDCoD. Global, regional, and national age-sex specific mortality for 264 causes of death, 1980–2016: a systematic analysis for the Global Burden of Disease Study 2016. Lancet. 2017;390:1151–1210 10.1016/S0140-6736(17)32152-9 PMC5605883 28919116

[6] Zeng Y, Hesketh T. The effects of China’s universal two-child policy. Lancet. 2016;388:1930–8. 10.1016/S0140-6736(16)31405-2 PMC5944611 27751400

[7] Cleary-Goldman J, Malone FD, Vidaver J, Ball RH, Nyberg DA, Comstock CH (2005). Impact of maternal age on obstetric outcome. Obstet Gynecol.

[8] Lisonkova S, Potts J, Muraca GM, Razaz N, Sabr Y, Chan WS (2017). Maternal age and severe maternal morbidity: A population-based retrospective cohort study. PLoS Med.

[9] Meng L, Huang J, Dong J (2018). Assessment of rural ecosystem health and type classification in Jiangsu province, China. The Science of the total environment.

[10] Maternal and Child Health Department National Health and Family Planning Commission of China . National maternal and child health report 2014. National Health and Family Planning Commission of China; Beijing: 2015.

[11] Gan XL, Hao CL, Dong XJ, Alexander S, Dramaix MW, Hu LN (2014). Provincial maternal mortality surveillance systems in China. Biomed Res Int.

[12] WHO Health statistics and information systems: maternal mortality ratio (per 100 000 live births) http://www.who.int/healthinfo/statistics/indmaternalmortality/en. Accessed 5 Apr 2016.

[13] Liu S, Wu X, Lopez AD, Wang L, Cai Y, Page A (2016). An integrated national mortality surveillance system for death registration and mortality surveillance, China. Bull World Health Organ.

[14] Jiangsu Bureau of Statistics of China. Jiangsu statistical yearbook 2018. Jiangsu Bureau of Statistics of China; Nanjing: 2018

[15] Jiangsu Provincial Health Commission. Jiangsu Health statistical yearbook 2018. Jiangsu Provincial Health Commission; Nanjing: 2018

[16] Liang J, Dai L, Zhu J, Li X, Zeng W, Wang H (2011). Preventable maternal mortality: geographic/rural-urban differences and associated factors from the population-based Maternal Mortality Surveillance System in China. BMC Public Health.

[17] Berg CJ, Harper MA, Atkinson SM, Bell EA, Brown HL, Hage ML (2005). Preventability of pregnancy-related deaths: results of a state-wide review. Obstet Gynecol.

[18] World Health Organization (2004). Beyond the numbers: reviewing maternal deaths and complications to make pregnancy safer.

[19] Hogan MC, Foreman KJ, Naghavi M, Ahn SY, Wang M, Makela SM (2010). Maternal mortality for 181 countries, 1980–2008: a systematic analysis of progress towards Millennium Development Goal 5. Lancet.

[20] United Nations, Ministry of Foreign Affairs. China’s progress towards the millennium development goals 2008 report. Accessed 6 Oct 2009

[21] Li J, Luo C, Deng R, Jacoby P, de Klerk N. Maternal mortality in Yunnan, China: recent trends and associated factors. BJOG. 2007;114:865–874 10.1111/j.1471-0528.2007.01362.x 17506792

[22] China MOH (2009). National Health Statistic Yearbook 2009.

[23] Say L, Raine R (2007). A systematic review of inequalities in the use of maternal health care in developing countries: examining the scale of the problem and the importance of context. Bull World Health Organ.

[24] Hesketh T, Zhou X, Wang Y (2015). The End of the One-Child Policy: Lasting Implications for China. JAMA.

[25] Kenny LC, Lavender T, McNamee R, O’Neill SM, Mills T, Khashan AS. Advanced maternal age and adverse pregnancy outcome: evidence from a large contemporary cohort. PLoS ONE. 2013;8:e56583. 10.1371/journal.pone.0056583 PMC3577849 23437176

[26] Khandwala YS, Baker VL, Shaw GM, Stevenson DK, Lu Y, Eisenberg ML (2018). Association of paternal age with perinatal outcomes between 2007 and 2016 in the United States: population based cohort study. BMJ.

[27] Laopaiboon M, Lumbiganon P, Intarut N, Mori R, Ganchimeg T, Vogel JP, et al. Advanced maternal age and pregnancy outcomes: a multicountry assessment. BJOG : an international journal of obstetrics and gynaecology. 2014;121(Suppl 1):49–56. 10.1111/1471-0528.12659 24641535

[28] Tang S, Meng Q, Chen L, Bekedam H, Evans T, Whitehead M (2008). Tackling the challenges to health equity in China. Lancet.

[29] Haixia Lu. Rational thoughts on imbalanced allocation of China’s rural basic health resources. Chinese Health Economics. 2009;28:38–42 (in Chinese).

[30] Luo R, Yang Qi, Jin Xi (2007). Analysis on medical and health personnel collocation of WCH of province and prefecture and county. Maternal & Child health care of China.

[31] Nagaya K, Fetters MD, Ishikawa M, Kubo T, Koyanagi T, Saito Y (2000). Causes of maternal mortality in Japan. JAMA.

[32] De Lange TE, Budde MP, Heard AR, Tucker G, Kennare R, Dekker GA (2008). Avoidable risk factors in perinatal deaths: a perinatal audit in South Australia. Aust N Z J Obstet Gynaecol.

[33] Ozumba BC, Nwogu-Ikojo EE (2008). Avoidable maternal mortality in Enugu. Nigeria Public health.

[34] Alves SV (2007). Maternal mortality in Pernambuco, Brazil: what has changed in ten years?. Reprod Health Matters.

