# Supplementary material for: Maternal mortality ratio in Jiangsu Province, China: recent trends and associated factors
Source: BMC Pregnancy Childbirth. 2021 Jun 25;21:447. doi: 10.1186/s12884-021-03897-0 (PMC8235612; doi:10.1186/s12884-021-03897-0)
Supplement: Supplementary file 1 — Additional file 1: Figure S1. Trends of MMR in Jiangsu province from 2013 to 2018. Figure S2. The percentage of multiparous mothers and advanced maternal age (≥35) of all pregnant women and maternal deaths from 2017 to 2018 in Jiangsu province. ***: P<0.001. The label under the figure: Percentage, % (95% confidence interval (CI)). Table S1. Objective risk factors for maternal death were different between the two groups. [file 12884_2021_3897_MOESM1_ESM.doc]

Title page

Maternal Mortality Ratio in Jiangsu Province, China: recent trends and associated factors

Donghua Li^1†^, Chengxiao Yu^2,3†^, Ci Song^2,3†^, Weiqing Ning^1^, Yan Xu^4^, Huan Ge^1^, Song Lin^1^, Wenjie Zhou^1^, Yajun Lu^1^, Xudong Wang^1^, Zhibin Hu^2,3^, Yuan Lin^5*^, Jie Wu^1*^.

**Figure S1. Trends of MMR in Jiangsu province from 2013 to 2018.**


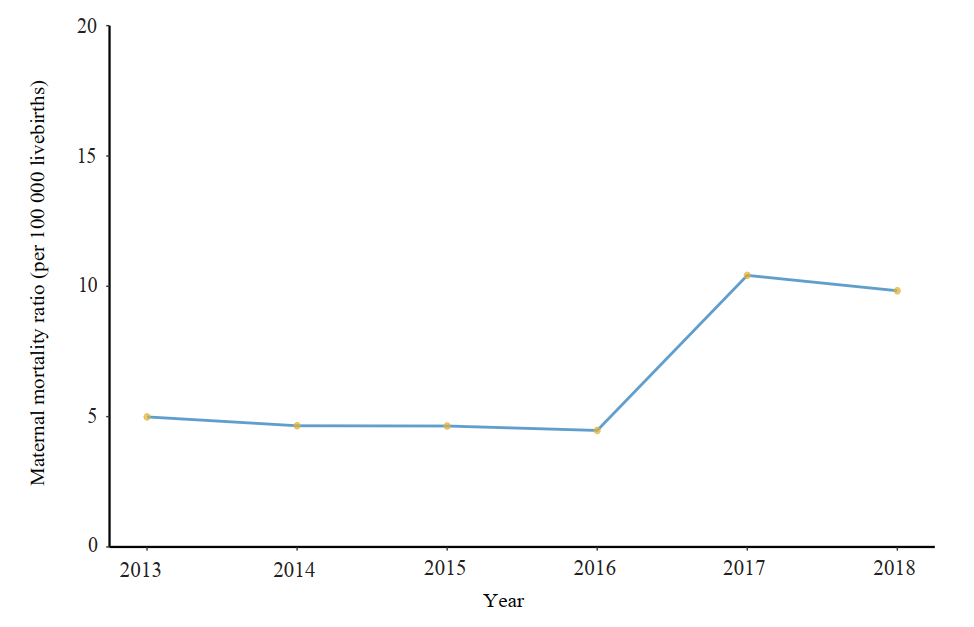


**Figure S2. The percentage of multiparous mothers and advanced maternal age (≥35) of all pregnant women and maternal deaths from 2017 to 2018 in Jiangsu province.** ***: *P*<0.001. The label under the figure: Percentage, % (95% confidence interval (CI)).


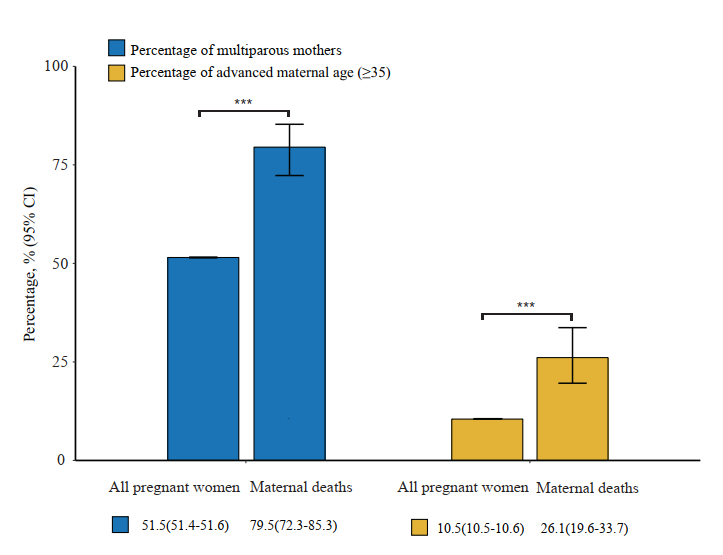


**Table S1. Objective risk factors for maternal death were different between the two groups.**

|  | **Overall^†^** | **Group1^†^** | **Group2^†^** | ***P**** |
| --- | --- | --- | --- | --- |
| Permanent population | 125.458 ± 93.754 | 113.523 ± 64.706 | 151.714 ± 136.433 | 2.45E-01 |
| Rate of population growth | -0.0002 ± 0.0078 | -0.0011 ± 0.0059 | 0.0018 ± 0.0107 | 2.58E-01 |
| GDP per capital, ¥10000 | 10.481 ± 4.555 | 8.263 ± 3.054 | 15.361 ± 3.349 | **9.31E-12** |
| Primary industry of GDP, ¥100 million | 61.286 ± 37.054 | 72.632 ± 34.644 | 36.326 ± 29.704 | **1.43E-04** |
| Secondary industry of GDP, ¥100 million | 637.148 ± 692.896 | 444.088 ± 347.750 | 1061.878 ± 1020.803 | **1.54E-02** |
| Tertiary Industry of GDP, ¥100 million | 679.919 ± 728.261 | 440.701 ± 370.245 | 1206.199 ± 1012.062 | **3.48E-03** |
| Rate of health care card | 0.988 ± 0.007 | 0.988 ± 0.008 | 0.986 ± 0.006 | 2.70E-01 |
| Rate of prenatal examination | 0.987 ± 0.006 | 0.988 ± 0.006 | 0.986 ± 0.005 | 1.87E-01 |
| Rate of five prenatal examinations | 0.969 ± 0.024 | 0.965 ± 0.028 | 0.977 ± 0.012 | **2.48E-02** |
| Rate of Early pregnancy examination | 0.954 ± 0.039 | 0.947 ± 0.044 | 0.969 ± 0.019 | **6.65E-03** |
| Rate of postpartum visit | 0.977 ± 0.015 | 0.975 ± 0.017 | 0.981 ± 0.008 | 6.29E-02 |
| Rate of maternal system management | 0.947 ± 0.042 | 0.940 ± 0.046 | 0.964 ± 0.026 | **9.23E-03** |
| Rate of delivery in maternal and child health hospital | 0.175 ± 0.185 | 0.119 ± 0.145 | 0.298 ± 0.207 | **1.80E-04** |
| Rate of delivery in public general hospital or maternal and child health hospital | 0.631 ± 0.238 | 0.560 ± 0.246 | 0.789 ± 0.114 | **3.50E-06** |
| Rate of cesarean section rate | 0.457 ± 0.096 | 0.467 ± 0.111 | 0.436 ± 0.047 | 1.18E-01 |
| Rate of moderate to severe anemia | 0.010 ± 0.012 | 0.011 ± 0.013 | 0.007 ± 0.008 | 1.61E-01 |
| Rate of high risk for prenatal screening | 0.045 ± 0.038 | 0.030 ± 0.019 | 0.078 ± 0.048 | **2.50E-04** |
| Rate of maternal at risk | 0.429 ± 0.132 | 0.387 ± 0.131 | 0.520 ± 0.078 | **5.38E-06** |
| Rate of low birth weight | 0.025 ± 0.015 | 0.019 ± 0.007 | 0.039 ± 0.017 | **8.84E-05** |
| Rate of high birth weight | 0.085 ± 0.027 | 0.087 ± 0.032 | 0.079 ± 0.010 | 1.46E-01 |
| Rate of preterm birth | 0.034 ± 0.021 | 0.024 ± 0.011 | 0.055 ± 0.022 | **3.09E-06** |
| Rate of stillbirth | 0.002 ± 0.001 | 0.002 ± 0.001 | 0.003 ± 0.002 | 2.20E-01 |

* Welch's t-test was applied for comparison of differences between two groups.

^†^ Mean ± SD (standard deviation).
